# The Postprandial Anti-Hyperglycemic Effect of Pyridoxine and Its Derivatives Using In Vitro and In Vivo Animal Models

**DOI:** 10.3390/nu10030285

**Published:** 2018-02-28

**Authors:** Hyuk Hwa Kim, Yu-Ri Kang, Jung-Yun Lee, Hung-Bae Chang, Ki Won Lee, Emmanouil Apostolidis, Young-In Kwon

**Affiliations:** 1 Department of Food and Animal Biotechnology, Seoul National University, 08826 Seoul, Korea; biohugh@gmail.com (H.H.K.); kiwon@snu.ac.kr (K.W.L.); 2 Department of Food and Nutrition, Hannam University, 34049 Daejeon, Korea; kang0606@hotmail.com (Y.-R.K.); seembeeks@hanmail.net (J.-Y.L.); 3 Department of Bio Quality Control, Korea Bio Polytechnic, 32943 Chungnam, Korea; hbchang@kopo.ac.kr; 4 Department of Chemistry and Food Science, Framingham State University, Framingham, MA 01701, USA; eapostolidis@framingham.edu

**Keywords:** pyridoxine, anti-hyperglycemia, postprandial, α-glucosidase, inhibition

## Abstract

In the current study, we investigated the inhibitory activity of pyridoxine, pyridoxal, and pyridoxamine, against various digestive enzymes such as α-glucosidases, sucrase, maltase, and glucoamylase. Inhibition of these enzymes involved in the absorption of disaccharide can improve post-prandial hyperglycemia due to a carbohydrate-based diet. Pyridoxal (4.14 mg/mL of IC_50_) had the highest rat intestinal α-glucosidase inhibitory activity, followed by pyridoxamine and pyridoxine (4.85 and 5.02 mg/mL of IC_50_, respectively). Pyridoxal demonstrated superior inhibition against maltase (0.38 mg/mL IC_50_) and glucoamylase (0.27 mg/mLIC_50_). In addition, pyridoxal showed significant higher α-amylase inhibitory activity (10.87 mg/mL of IC_50_) than that of pyridoxine (23.18 mg/mL of IC_50_). This indicates that pyridoxal can also inhibit starch hydrolyzing by pancreatic α-amylase in small intestine. Based on these in vitro results, the deeper evaluation of the anti-hyperglycemic potential of pyridoxine and its derivatives using Sprague-Dawley (SD) rat models, was initiated. The post-prandial blood glucose levels were tested two hours after sucrose/starch administration, with and without pyridoxine and its derivatives. In the animal trial, pyridoxal (*p* < 0.05) had a significantly reduction to the postprandial glucose levels, when compared to the control. The maximum blood glucose levels (C*max*) of pyridoxal administration group were decreased by about 18% (from 199.52 ± 22.93 to 164.10 ± 10.27, *p* < 0.05) and 19% (from 216.92 ± 12.46 to 175.36 ± 10.84, *p* < 0.05) in sucrose and starch loading tests, respectively, when compared to the control in pharmacodynamics study. The pyridoxal administration significantly decreased the minimum, maximum, and mean level of post-prandial blood glucose at 0.5 h after meals. These results indicate that water-soluble vitamin pyridoxine and its derivatives can decrease blood glucose level via the inhibition of carbohydrate-hydrolyzing and absorption-linked enzymes. Therefore, pyridoxal may have the potential to be used as a food ingredient for the prevention of prediabetes progression to type 2 diabetes.

## 1. Introduction

Type 2 Diabetes Mellitus (T2DM), a common disorder of glucose metabolism, is linked to insulin resistance and high calorie diets. T2DM is associated with changes in dietary pattern towards high calorie sweetened foods with disaccharides such as maltose and sucrose [1]. T2DM accounts for the vast majority of diabetes cases in adults [2], and expecting to reach 366 million diabetes patients by the year 2030 [3]. Specifically in the United States, it is expected that 1/3 of all adults will have diabetes by 2030 [4].

High blood glucose levels are typical in type 2 diabetes patients, and more specifically this is characterized by a postprandial increase of glucose levels following food consumption, due to digestion of carbohydrates by pancreatic α-amylase and small intestinal α-glucosidases, resulting in elevated liberation of glucose [5]. A strategy to reduce high blood glucose levels following meal administration, is through the inhibition of enzymes that hydrolyze carbohydrates, in the small intestine [6,7]. Therefore, commercial available α-glucosidase inhibitors, Acarbose^®^ (Bayer AG, Leverkusen, Germany), Voglibose^®^ (Takeda, Tokyo, Japan), and Miglitol^®^ (Bayer AG, Leverkusen, Germany) are generally prescribed either alone, or in combination with insulin sensitizers in T2DM patients, clinically.

Pyridoxine (Vitamin B6) has been used for managing normal cognitive function and in lowering the incidence of coronary heart disease (CHD) among the seniors [8,9,10]. In addition, vitamin B6 administration has been shown to decrease complications of diabetes and the incidence of neurodegenerative diseases [11]. Reduced levels of vitamin B6 have been associated with the existence of both type 1 and type 2 diabetic diabetes [11,12,13]. Other studies have shown that B6 administration reduces the clinical symptoms of retinopathy and neuropathy in diabetic patients [14,15]. When the effect of vitamin B6 was evaluated on STZ-mice, a blood glucose level reduction was observed [16], and in type 2 diabetes patients HbA1c levels were reduced [17]. Other in vitro evaluations of the vitamin B6 derivative pyridoxamine have indicated the effect on the inhibition of formation of AGE products [18].

However, pyridoxine and its derivatives have not been thoroughly evaluated for their possible blood glucose lowering effect and there is no study suggesting the exact mechanism of vitamin B6 for this specific health benefit. Therefore, the aim of this study is to investigate mode of action and effect of pyridoxine and its derivatives on postprandial hyperglycemia. To determine the above, inhibitory activities of pyridoxine, pyridoxal, and pyridoxamine are investigated against the α-glucosidase and α-amylase (anti-hyperglycemia potential). Anti-hyperglycemic effect of these compounds was also evaluated in Sprague-Dawley (SD) rat models. This work will contribute towards the understanding of the activity and the mechanism of action of vitamin B6 and its derivatives, specifically towards the management and prevention of type 2 diabetes. 

## 2. Materials and Methods 

### 2.1. Materials

Water-soluble pyridoxine and its derivatives was used from reagent companies in Sigma-Aldrich (Sigma-Aldrich Co. LLC., St. Louis, MO, USA), Daejung (Daejung Chemicals & Metals Co., Ltd., Gyeonggi-do, Korea), Duksan (Duksan Pure Chemical Co., Ltd., Gyeonggi-do, Korea), and Junsei (Junsei Chemical Co., Ltd., Tokyo, Japan). Rat intestinal acetone powder, *p*-nitrophenyl-α-d-glucopyranoside (*p*NPG), porcine pancreatic α-amlyase enzyme powder, starch, sucrose, and maltose were purchased from Sigma-Aldrich (St. Luis, MO, USA). Unless noted, all chemicals were purchased from Sigma-Aldrich (St. Luis, MO, USA).

### 2.2. Carbohydrate-Hydrolyzing Enzyme Inhibition Assay

#### 2.2.1. Rat Intestinal α-Glucosidase Inhibition Assay

The rat intestinal α-glucosidase assay was administered as per the method of Kwon et al. [19] with slight modification. A total of 1 g of rat-intestinal acetone powder was suspended in 3 mL of 0.1 M sodium phosphate buffer (pH 6.9), and the suspension was sonicated 12 times for 30 s at 4 °C. After centrifugation (10,000× *g*, 30 min, 4 °C), the resulting supernatant was used for the assay. Sample solution (50 μL) and 0.1 phosphate buffer (pH 6.9, 100 μL) containing α-glucosidase (Sigma-Aldrich, St. Luis, MO, USA) solution (1.0 U/mL) was incubated at 37 °C for 10 min. After pre-incubation, 5 mM *p*-nitrophenyl-α-d-glucopyranoside solution (50 μL) in 0.1 M phosphate buffer (pH 6.9) was added to each well at timed intervals. The reaction mixtures were incubated at 37 °C for 30 min. Before and after incubation, absorbance was read at 405 nm and compared to a control, which had 50 μL of buffer solution in place of the extract by micro-plate reader (SUNRISE; Tecan Trading AG, Salzburg, Austria). The α-glucosidase inhibitory activity was expressed as inhibition % and was calculated as follows:

$$\mathrm{Inhibition}\left(\text{\%}\right)=\left(\frac{\Delta A\;405\;\left(\mathrm{Control}\right)-\Delta A\;405\left(\mathrm{Extract}\right)}{\Delta A\;405\;\left(\mathrm{Control}\right)}\right)\times 100$$

#### 2.2.2. Porcine Pancreatic α-Amlyase Inhibition Assay

The porcine pancreatic α-amlyase inhibition assay was administered as per the method of Kwon et al. [20] Sample solution (200 μL) and 0.02 M sodium phosphate buffer (pH 6.9 with 0.006 M sodium chloride, 500 μL) containing α-amylase solution (0.5 mg/mL, 5.0 MU/mL) were incubated at 25 °C for 10 min. After preincubation, 500 μL of a 1% starch solution in 0.02 M sodium phosphate buffer was added. The reaction mixture was then incubated at 25 °C for 10 min. The reaction was stopped with 1.0 mL of dinitrosalicylic acid (DNS). The reaction mixture was then incubated in a boiling water bath for 5 min and cooled to room temperature. The reaction mixture at 540 nm with ELISA microplate reader (SUNRISE; Tecan Trading AG, Saltzburg, Austria).

#### 2.2.3. Maltase, Sucrase, and Glucoamylase Inhibition Assay

The crude enzyme solution prepared from rat intestinal acetone powder Sigma-Aldrich Co. (St. Louise, MO, USA) was used as the small intestinal maltase, sucrose, and glucoamylase, showing specific activities of 0.70, 0.34, and 0.45 units/mL, respectively. Rat intestinal acetone powder (1.0 g) was suspended in 3 mL of 0.1 M sodium phosphate buffer, and the suspension was sonicated 12 times for 30 s at 4 °C. After centrifugation (10,000× *g*, 30 min, 4 °C), the resulting supernatant was used for the assay. Maltase, sucrose, and glucoamylase inhibitory activity were assay by modifying a method developed by Dahlqvist [21]. The inhibitory activity was determined by incubating a solution of an enzyme (50 μL), 0.1 M phosphate buffer (pH 7.0, 100 μL) containing 0.4 mg/mL sucrose or maltose or 1% soluble starch, and a solution (50 μL) with various concentrations of sample solution (between 0.05 and 1.0 mM) at 37 °C for 30 min. The reaction mixture was heated in a boiling water bath to stop the reaction for 10 min, and the amount of liberated glucose was then measured by the glucose oxidase [22]. The inhibitory activity was calculated from the formula as follows. Inhibition (%) = (C − T)/C × 100, where C is the enzyme activity without inhibitor, and T is the enzyme activity with inhibitor.

### 2.3. The Sucrose/Starch Loading Test

Effect on hyperglycemia induced by carbohydrate loads in Sprague-Dawley (SD) rats was determined by an inhibitory action of water-soluble vitamins and Acarbose on postprandial hyperglycemia. All animal procedures were approved by Institutional Animal Care and Use Committee (IACUC) of the Hannam University (Approval number: HNU2017-003). Four-week-old male SD rats were purchased from Raon Bio Co. (Gyeonggi-do, Korea) and fed a solid diet (Raon Bio Co., Gyeonggi-do, Korea) for one week. The rats were housed in a ventilated room at 23 ± 2 °C with 50 ± 5% relative humidity, and under an alternating 12 h light/dark cycle. After 7 groups of 4 male SD rats (130~150 g) were fasted for 24 h, 2.0 g/kg of sucrose or starch were orally administrated concurrently with 0~100 mg/kg inhibitors (water-soluble vitamins and Acarbose). The blood samples were then taken from the tail after administration and blood glucose levels were measured at 0, 0.5, 1, 2, and 3 h. The glucose level in blood was determined by glucose oxidase method and compared with that of the control group, which had not taken the inhibitors.

### 2.4. Statistical Analysis

All data are presented as mean ± Standard deviation (SD). Statistical analysis was carried out using statistical package SPSS 10 (Statistical Package for Social Science; SPSS Inc., Chicago, IL, USA), and the significance of each group was verified with the analysis of one-way analysis of variance (ANOVA) followed by Duncan’s test of *p* < 0.05.

## 3. Results

### 3.1. Rat Intestinal α-Glucosidase Inhibitory Activity of Water-Soluble Vitamins

α-Glucosidase inhibitors, such as Acarbose^®^ and Voglibose^®^, delay the digestion of oligosaccharide and disaccharide to monosaccharide by inhibiting α-glucosidases on the small intestinal brush-border, and reduce the rate of glucose absorption [6]. Inhibition of these enzymes involved in the absorption of disaccharide can improve post-prandial hyperglycemia due to the consumption of carbohydrate-based diet. As a result, administration of such inhibitors prior to meal consumption result in reduced postprandial blood glucose concentrations. 

**Figure 1:**
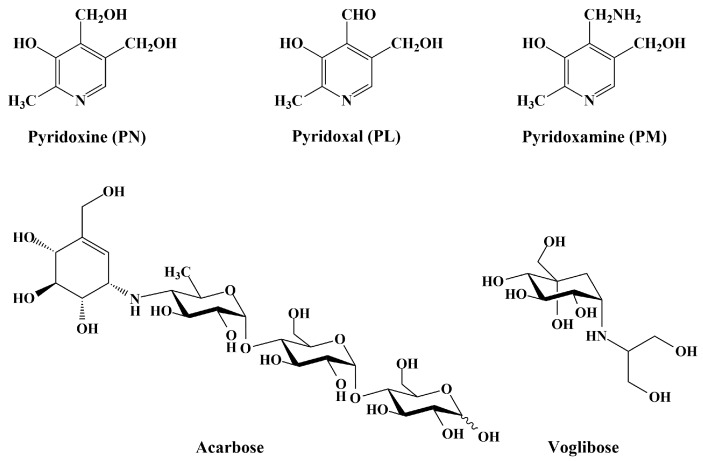
Structure of pyridoxine and its derivatives (pyridoxal and pyridoxamine) and commercial α-glucosidase inhibitors (Acarbose^®^ and Voglibose^®^).

**Figure 2:**
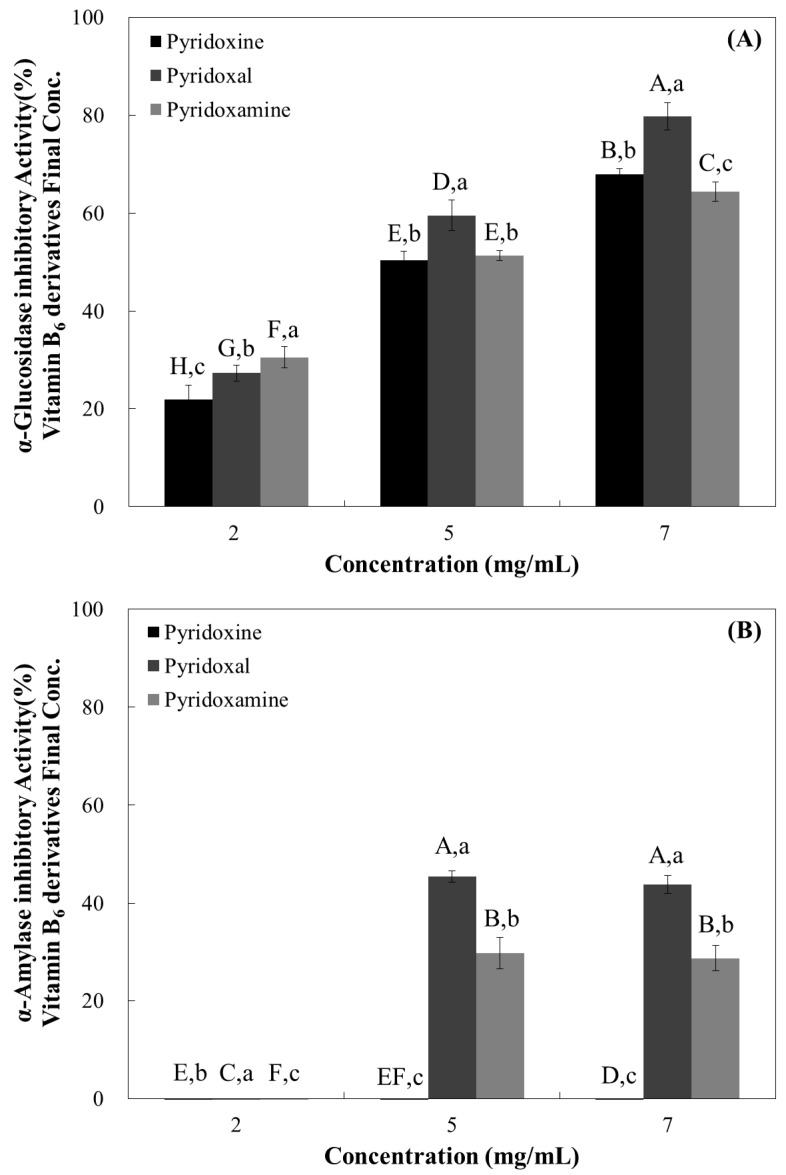
Dose-dependent changes in rat intestinal α-glucosidase (**A**) and porcine pancreatic α-amylase inhibitory activities (**B**) (% inhibition) of pyridoxine, pyridoxal, and pyridoxamine. The results are expressed as mean ± S.D. with three independent experiments in triplicate. Different corresponding letters indicate significant differences at *p* < 0.05 by Duncan’s test. The first letters in uppercase (^A–H^) indicate significant differences among all samples. The second letters in lowercase (^a–c^) are different among types of vitamin within the same concentration.

To screen the α-glucosidase inhibitory effects of vitamin B6 and its derivatives, we examined α-glucosidase activity using rat acetone powder (Figure 1). Pyridoxal exhibited the highest inhibitory effect among the tested compounds, resulting in a 79.83% inhibition at the highest tested dose (7 mg/mL) (Figure 2). Pyridoxamine and pyridoxine appeared to have similar inhibitory activities, but showed significantly less activity compared with pyridoxal (Figure 2). When the IC_50_ values were calculated, we observed that pyridoxal had the lowest value (4.15 mg/mL), while pyridoxine had the highest (5.02 mg/mL) (Table 1).

Our observations suggest that pyridoxal has the highest inhibitory potential against α-glucosidase while pyridoxine had the lowest (Figure 2 and Table 1).

### 3.2. Porcine Pancreatic α-Amlyase Inhibition Assay

When the pancreatic α-amlyase inhibitory activity was evaluated, we observed no inhibitory effect at 2 mg/mL concentrations in all samples. Pyridoxine showed no inhibitory effects, even at the highest tested dose (7 mg/mL) (Figure 2). Pyridoxal and pyridoxamine showed α-amylase inhibitory effects at the two highest tested doses (5 and 7 mg/mL) with pyridoxal appearing to have a higher inhibitory effect (Figure 2). When the IC_50_ values were calculated, we observed that pyridoxal had the lowest value (12.92 mg/mL), while pyridoxine had the highest (23.17 mg/mL) (Table 1).

Our observations are similar to our previous results, suggesting that pyridoxal has the highest inhibitory potential against α-amylase, while pyridoxine had the lowest (Figure 2 and Table 1).

### 3.3. Maltase, Sucrase, and Glucoamylase Inhibition Assay

Since α-glucosidase inhibitory effects of the vitamin B6 group was shown, we examined whether vitamin B6 group could selectively inhibit enzymes in the small intestine, namely sucrase, maltase, and glucoamylase. 

**Figure 3:**
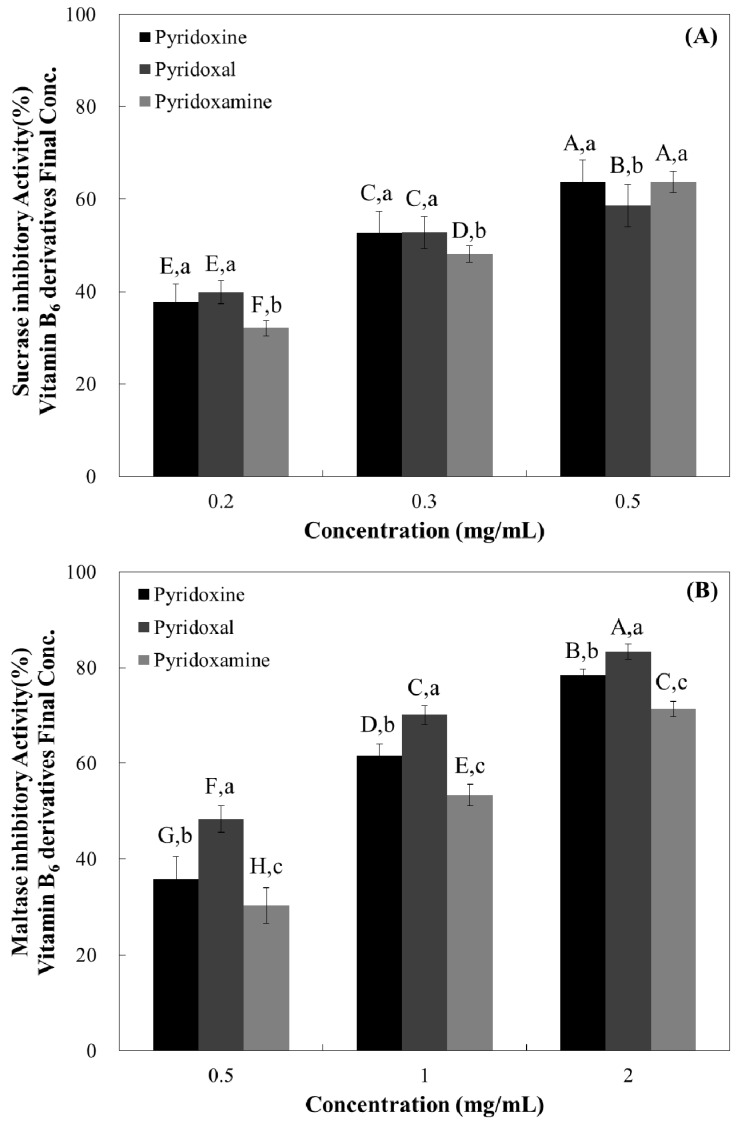

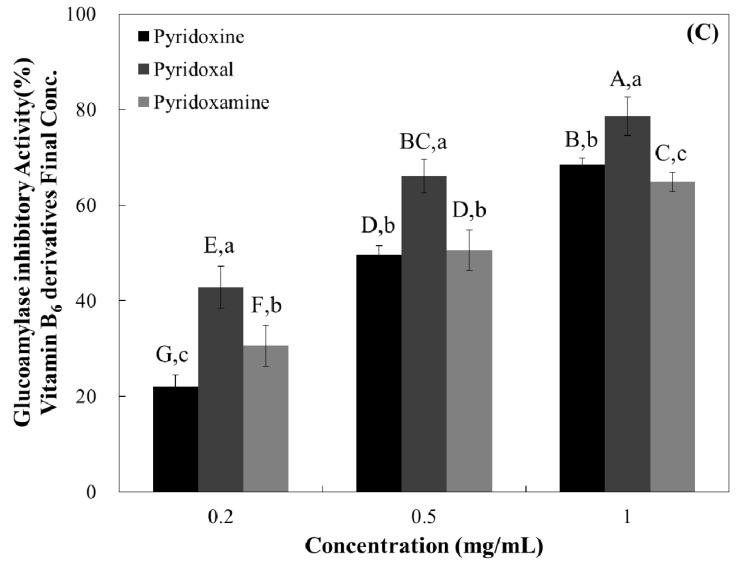
Dose-dependent changes in rat intestinal α-glucosidases such as sucrase (**A**); maltase (**B**); and glucoamylase inhibitory activities (**C**) (% inhibition) of pyridoxine, pyridoxal, and pyridoxamine. The results are expressed as mean ± S.D. with three independent experiments in triplicate. Different corresponding letters indicate significant differences at *p* < 0.05 by Duncan’s test. The first letters in uppercase (^A–H^) indicate significant differences among all samples. The second letters in lowercase (^a–c^) are different among types of vitamin within the same concentration.

The sucrase inhibitory effect of the tested compounds revealed that all of them had similar inhibitory effects (Figure 3, Table 1). All samples demonstrated maltase inhibitory activity in a dose-dependent manner (Figure 3) and pyridoxal resulted in the highest maltase inhibitory activity at all tested doses (0.5 mg/mL—48.39%, 1 mg/mL—70.10%, and 2 mg/mL—83.29%) (Figure 3).

Similar to maltase inhibitory activity, all tested vitamin B6 structures resulted in dose-dependent glucoamylase inhibition (Figure 3), and pyridoxal had the highest inhibitory effect at all tested doses (42.84% at 0.2 mg/mL, 66.07% at 0.5 mg/mL, and 78.59% at 1 mg/mL) (Figure 3). 

Based on these dose-dependent results, half maximal concentration (IC_50_) of samples in vitro system was shown in Table 1. Pyridoxal yielded to the lower IC_50_ value for maltase and glucoamylase (0.38 and 0.27 mg/mL, respectively), suggesting higher inhibition potential. Against sucrase all tested samples yielded similar and not significant different IC_50_ values (Table 1), suggesting that all tested compounds have similar inhibitory potential against this carbohydrate-hydrolyzing enzyme.

### 3.4. Starch/Sucrose Loading Test

A sucrose loading test using SD rat models was established, during which we evaluated the changes in the postprandial blood glucose levels, as described in the materials and methods.

**Figure 4:**
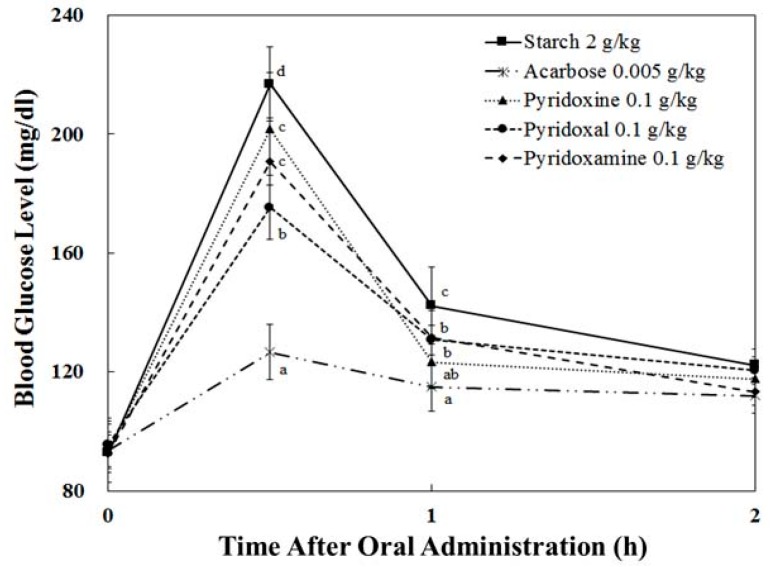
Postprandial blood glucose-lowering effects of pyridoxine, pyridoxal, and pyridoxamine in the starch loading test. After fasting for 24 h, six-week-old, male SD rats were orally administered with a starch solution (2.0 g/kg) with or without samples (pyridoxine, pyridoxal, pyridoxamine, and Acarbose). Each point represents mean ± S.D. (*n* = 5). The letters in lowercase (^a–d^) indicate significant differences (*p* < 0.05) at 0.5 h and 1.0 h, respectively, via Duncan’s test.

In the pyridoxal-treated group with starch, the blood glucose level was 175.36 ± 10.84 mg/dL at 30 min after administration. Compared to the pyridoxine-treated group (201.78 ± 18.99 mg/dL) and starch group (216.92 ± 12.46 mg) (Figure 4), pyridoxal-treated group suppresses the rising of plasma glucose level by 26.41 mg/dL, and 41.56 mg/dL, respectively, after administration. At 30 min after administration, we found that the pyridoxal-treated group showed the lowest increase in blood glucose level (79.78 ± 10.84 mg/dL), which was 27.91 mg/dL less than that of the pyridoxine-treated group (107.69 ± 18.99 mg/dL) and 44.45 mg/dL less than that of the starch group (124.23 ± 12.46 mg/dL).

**Figure 5:**
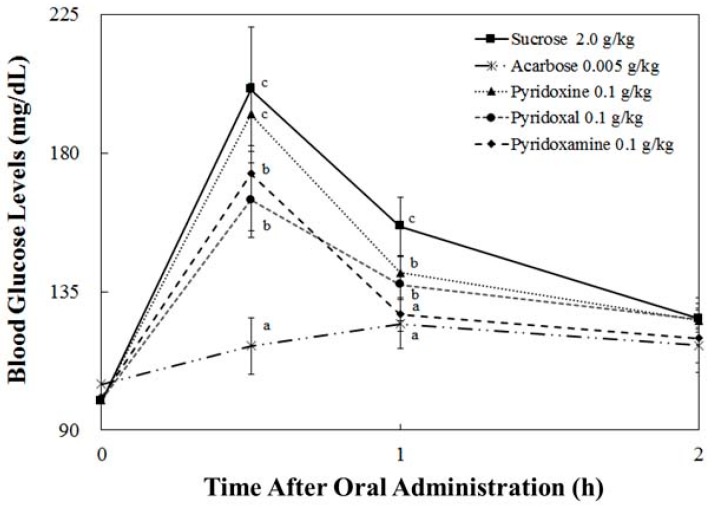
Postprandial blood glucose-lowering effects of pyridoxine, pyridoxal, and pyridoxamine in the sucrose loading test. After fasting for 24 h, six-week-old, male SD rats were orally administered with a sucrose solution (2.0 g/kg) with or without samples (pyridoxine, pyridoxal, pyridoxamine, and Acarbose). Each point represents mean ± S.D. (*n* = 5). The letters in lowercase (^a–c^) indicate significant differences (*p* < 0.05) at 0.5 h and 1.0 h, respectively, via Duncan’s test.

Among the three tested compounds, change of blood glucose level in the pyridoxal-treated group with sucrose was increased by 64.88 ± 12.06 mg/dL at 30 min after administration (Figure 5). This is lower than the pyridoxine-treated group (92.86 ± 10.10 mg/dL) and the sucrose group (101.27 ± 20.18 mg/dL) by 27.98 mg/dL and 36/39 mg/dL, respectively. At 30 min after administration, we found that the blood glucose level of the pyridoxal-treated group was 164.74 ± 12.06 mg/dL, which was 27.76 mg/dL less than that of the pyridoxine-treated group (192.50 ± 10.10 mg/dL) and 35.89 mg/dL less than that of the sucrose-treated group (200.63 ± 20.18 mg/dL).

### 3.5. Pharmacodynamics Parameters

Pharmacodynamic parameters of the sucrose and starch loading test are shown in Table 2. In terms of T*max*, there is no significance between sucrose/starch and vitamin B6-treated groups. In contrast, vitamin B6-treated groups resulted in significantly decreased C*max* and AUC*t* values (both with sucrose and starch loading), but less effective than the Acarbose group. 

In sucrose loading, pyridoxal and pyridoxamine resulted to the highest reduction both in terms of C*max* and AUC (Table 2). In the case of starch loading, pyridoxal resulted in the greatest reduction of C*max*, while all tested compounds had similar AUC values (Table 2). In terms of absolute values, the maximum blood glucose levels (C*max*) of the pyridoxal administration group were decreased by about 18% (from 199.52 ± 22.93 to 164.10 ± 10.27, *p* < 0.05) and 19% (from 216.92 ± 12.46 to 175.36 ± 10.84, *p* < 0.05) in the sucrose and starch loading tests, respectively, when compared to the control in the pharmacodynamics study.

Our findings suggest that vitamin B6 and its derivatives can inhibit the breakdown of disaccharide to monomer in brush-border and suppress the entrance of blood glucose into the bloodstream.

## 4. Discussion

In this manuscript, we are presenting the first report of vitamin B6 and its derivatives, for the potential management of type 2 diabetes, via the inhibition of small-intestinal α-glucosidases. Our significant in vitro and in vivo findings indicate that pyridoxine (vitamin B6) has α-glucosidase inhibitory activity, resulting in reduced postprandial blood glucose levels following sucrose and starch ingestion. 

The purpose of this research was to evaluate the mode of action and type 2 diabetes-relevant effect of vitamin B6 and its derivatives. More specifically, we evaluated the effect on in vivo postprandial hyperglycemia via the in vitro inhibition of carbohydrate-hydrolyzing enzymes. Vitamin B6 (pyridoxine, pyridoxal, and pyridoxamine) forms are readily absorbed by passive diffusion in the jejunum and ileum [23], while alpha-glucosidases are expressed and located in the duodenum [24]. Our studies suggest that the blood glucose lowering effect of pyridoxine is possibly due to the α-glucosidase inhibitory activities. Furthermore, our studies show that pyridoxine, one of essential vitamins has high activity of enzyme inhibition (on sucrase, maltase, and glucoamylase). These results could be attributed to the structural similarities between pyridoxine and Acarbose and Voglibose, the pharmacological agents used for the inhibition of carbohydrate-hydrolyzing enzymes (Figure 1). Compared to Acarbose, pyridoxine resulted in an enhanced inhibitory effect against α-glucosidases, but the inhibitory effect against α-amylase was significantly reduced, suggesting fewer side-effects. When the postprandial blood glucose levels in adult, normal SD rats were measured, we observed that pyridoxine administration resulted in a significant reduction in post-prandial blood glucose levels after 30 min (Figure 4 and Figure 5). More specifically, we observed a blood glucose reduction around 18% at 30 min when pyridoxal administration was compared to control. Taking into consideration that pyridoxine is present in a wide variety of food products, knowledge of this additional health benefit of pyridoxine can assist in the development of efficacious anti-hyperglycemia supplements and food products. 

## Figures and Tables

**Table 1:** The half maximal inhibitory concentration (IC_50_) of pyridoxine and its derivatives on rat intestinal α-glucosidase, sucrase, maltase, glucoamylase, and porcine pancreatic α-amylase activities.

IC_50_ (mg/mL)					
	Rat intestinal α-Glucosidase	Porcine Pancreatic α-Amylase	Rat Intestinal SUCRASE	Rat Intestinal Maltase	Rat Intestinal Glucoamylase
Pyridoxine	5.02 ± 0.05 ^a^	23.17 ± 0.29 ^a^	0.32 ± 0.03 ^a^	0.87 ± 0.08 ^a^	0.63 ± 0.04 ^a^
Pyridoxal	4.15 ± 0.17 ^b^	12.92 ± 1.22 ^c^	0.32 ± 0.03 ^a^	0.37 ± 0.13 ^b^	0.26 ± 0.08 ^b^
Pyridoxamine	4.85 ± 0.06 ^a^	14.93 ± 0.96 ^b^	0.35 ± 0.01 ^a^	1.08 ± 0.10 ^a^	0.60 ± 0.06 ^a^

The results are expressed as mean ± S.D. ^a–c^ Different letters indicate statistically significant differences between groups one-way ANOVA followed by Duncan’s test of p < 0.05.

**Table 2:** Pharmacodynamic (PD) parameters of control, pyridoxine, pyridoxal, pyridoxamine, and Acarbose in SD rats ingested with starch or sucrose.

	Groups	PD Parameters		
		C*max* (mg/dL)	T*max* (h)	AUC*t* (h∙mg/dL)
BGL (mg/dL)	Starch 2.0 g/kg	216.92 ± 11.58 ^d^	0.50 ± 0.00 ^a^	299.02 ± 12.10 ^c^
	Acarbose 0.005 g/kg	126.60 ± 9.44 ^a^	0.50 ± 0.00 ^a^	228.88 ± 13.09 ^a^
	Pyridoxine 0.1 g/kg	200.00 ± 20.89 ^c,d^	0.50 ± 0.00 ^a^	274.28 ± 15.62 ^b^
	Pyridoxal 0.1 g/kg	172.67 ± 11.45 ^b^	0.50 ± 0.00 ^a^	268.27 ± 8.80 ^b^
	Pyridoxamine 0.1 g/kg	191.54 ± 13.70 ^c^	0.50 ± 0.00 ^a^	275.10 ± 7.31 ^b^
BGL (mg/dL)	Sucrose 2.0 g/kg	199.52 ± 22.93 ^c^	0.50 ± 0.00 ^a^	303.57 ± 14.71 ^c^
	Acarbose 0.005 g/kg	126.92 ± 6.87 ^a^	0.86 ± 0.24 ^b^	237.94 ± 8.82 ^a^
	Pyridoxine 0.1 g/kg	192.48 ± 9.03 ^c^	0.50 ± 0.00 ^a^	290.64 ± 15.55 ^c^
	Pyridoxal 0.1 g/kg	164.10 ± 10.27 ^b^	0.50 ± 0.00 ^a^	272.99 ± 5.96 ^b^
	Pyridoxamine 0.1 g/kg	174.50 ± 18.29 ^b^	0.50 ± 0.00 ^a^	269.14 ± 13.40 ^b^

The results are expressed as mean ± S.D. The letters in lowercase (^a–d^) indicate statistically significant differences between groups one-way ANOVA followed by Duncan’s test of *p* < 0.05.
